# Growing around grief: the lived experience of parentally-bereaved young people in the UK

**DOI:** 10.1080/13576275.2025.2481279

**Published:** 2025-03-21

**Authors:** Mathilde Scott, Sam Quinn, Stephanie Chambers

**Affiliations:** a School of Social and Political Sciences, University of Glasgow, Glasgow, UK; b End of Life Studies Group, School of Social and Environmental Sustainability, University of Glasgow, Glasgow, UK; c MRC/CSO Social and Public Health Sciences Unit, University of Glasgow, Glasgow, UK

**Keywords:** Young people, dual process model, parental bereavement, health, support

## Abstract

More than 100 children per day in the UK experience the death of their parent before the age of 18. Parentally-bereaved young people (PBYP) face considerable hardships including detrimental health and wellbeing outcomes across their lifespan. This study’s aim was to explore the lived experience of PBYP in the UK to understand their experiences and needs. Nine semi-structured interviews were conducted with young people aged 18–25 in Scotland. Thematic analysis was carried out using the Dual Process Model (DPM) as an exploratory lens. Four themes were identified: grief experiences, cumulative stressors, valued support, and coping mechanisms and management strategies. The themes reflected the DPM’s components: loss-orientation, restoration-orientation and oscillation, but the DPM was not fully able to account for the wider social and interpersonal contextual factors discussed. The article proposes further research to explore extensions to the DPM to account for these factors. Practice recommendations include improved bereavement protocols, education for peers and educational staff, and bereavement support interventions.

## Introduction

### Background

In the UK, a parent dies every 22 minutes, and 41,000 children lose a parent every year (Childhood Bereavement Network, 2016). The death of a parent is a life-changing and often traumatic event for a child (Haine et al., 2008). Children who lose a parent face striking and unique challenges, including physical, psychological, social, health, cultural and educational hardships (McLaughlin et al., 2019). Parental bereavement has adverse impacts on long-term physical health (Luecken & Roubinov, 2012), including cardiovascular disease (Hollingshaus & Smith, 2015), and chronic illness (Raposa et al., 2014). It heightens the risk of poor mental health (Appel et al., 2018; Pham et al., 2018), criminal behaviour (Draper & Hancock, 2011), educational underachievement (Berg et al., 2014), social and emotional difficulties (Brent et al., 2012; Cerniglia et al., 2014), and maladaptive coping strategies, including self-medication, substance misuse and eating disorders (Brent et al., 2009; Hamdan et al., 2013; Hoeg et al., 2017).

Despite the significant impact early parental bereavement has, and the large number of young people who experience this, bereavement support provision in the UK is limited. Winston’s Wish, a leading UK childhood bereavement charity, found that childhood bereavement support provision in British schools was ‘patchy’ and incomprehensive (McLaughlin et al., 2019, p. 15). Within UK universities bereavement support for students is also neglected, without a collective, holistic approach to assisting bereaved students (Valentine & Woodthorpe, 2018). Half of PBYP perceive their needs to be unmet (Patterson & Rangganadhan, 2010). Providing effective support is imperative so that PBYP can adjust to their loss and altered life (McGachy, 2021).

Whilst there is a substantial body of literature which considers the adverse outcomes of parental loss throughout childhood, there are fewer empirical studies exploring PBYP’s lived experiences and needs (Patterson & Rangganadhan, 2010; Penny & Rice, 2012). Those that have been conducted report obstacles to receiving support, including not being offered or informed of support and services, discomfort in requesting support, and stigma (Chater et al., 2022). Other important unmet needs include opportunities to grieve, receive practical support and to have fun (Patterson & Rangganadhan, 2010).

For PBYP, heightened feelings of grief can occur during important moments throughout their lifetime including becoming a parent or passing a driving test (Chater et al., 2022), a phenomenon known as regrief (Oltjenbruns, 2001). Despite these challenges, as PBYP adjust, post-traumatic growth – positive transformations, ensuing adversities and trauma – may occur (Tedeschi & Calhoun, 1996). A recurring theme is the reported value of PBYP sharing with others who have also experienced parental loss (e.g. Brewer & Sparkes, 2011; Olsson et al., 2017; Patterson & Rangganadhan, 2010). Chater et al. (2022) found that following five years of bereavement, PBYP became increasingly resilient, and their life perspectives improved. Similarly, Apelian and Nesteruk (2017) reported that after eight years, PBYP felt as though they had suitably adjusted and gained ‘resilience’ and ‘personal growth’ (p. 92).

#### Grief models

Grief models have not been sufficiently applied to PBYP’s experiences and are founded on research predominantly conducted within adult populations who have lost a child or spouse (Damianakis & Marziali, 2012; Hawthorne et al., 2016). Staged-models of grief have historically focused on ‘grief work’, characterising grief as a linear or sequential process reaching a resolution (Bowlby & Parkes, 1970; Freud, 1917; Kubler-Ross, 1969; Stroebe, 1992). These more rigid models often fail to consider the dynamic processes that those who are grieving may go through (Osterweis et al., 1984; Silver & Wortman, 2007; Stroebe et al., 2017). In addition, these models lack empirical grounding, underestimating the complexity and value of coping mechanisms (Stroebe & Schut, 1999, 2010).

Contemporary theories have greater flexibility, centring around continued ties between the bereaved person and the person who has died and considering the ways those who are bereaved cope with loss. Continuing Bonds Theory focuses on healthy grieving, sustaining continued bonds with the deceased, and maintaining the deceased’s presence (Klass and; Goss, 1999; Klass et al., 1996). This can include maintaining bonds through dreams, preserving and sharing memories, symbolic objects and rituals (Christ, 2000; Christ et al., 2002; Hansen et al., 2015; Silverman & Nickman, 1996).

The Dual Process Model (DPM) establishes a process-oriented approach where oscillation between two significant coping processes and stressors after loss, loss-orientation and restoration-orientation, occur. The DPM outlines how people manoeuvre through life with both an ongoing feeling of loss and efforts to evade grief. Loss-orientation involves the bereaved person focusing on the loss and managing grief-associated emotions and reactions (Stroebe & Schut, 2010, p. 212). It is marked by grief intrusion – feeling the loss in daily life – the breaking of bonds, and the rejection of changes associated with restoration-tasks. Restoration-orientation refers to loss-related coping (Stroebe & Schut, 2010). The bereaved person adjusts to the daily life changes which accompany loss, including new challenges, tasks, identities, and responsibilities (Stroebe & Schut, 1999, 2010). Oscillation is a ‘dynamic and fluctuating’ process between the ‘confrontation and avoidance of different stressors’ (Stroebe & Schut, 1999, p. 215). This interchanging process between loss- and restoration-orientation enables the bereaved person to flexibly balance grief expression and loss adaptation, easing the grieving process. Three identified studies have applied the DPM to PBYP. Lundberg et al. (2018) used the DPM to detail PBYP’s loss- and restoration-oriented stressors and psychosocial wellbeing, and Patterson et al. (2021) presented the development of an intervention for PBYP to meet coping, support and respite needs. Larsen et al. (2023) presented two cases drawn from a Danish treatment programme for young adults experiencing complicated grief based around the DPM and other approaches, noting reported benefits of the intervention.

The aim of this study was to explore the lived experience of PBYP in the UK to understand their needs. Previous work in this area has focused on PBYP who experienced their loss much less recently than those who are the focus of this study. The study focuses on the experiences of those who are now ‘emerging adults’, that is young people aged 18–25 years. Emerging adulthood is a life stage where people have left adolescence but have not yet transitioned to young adulthood (Arnett, 2000). Previous bereavement research has not always considered the unique complexities faced by these young people (Morris et al., 2018), as they are frequently assumed to be ‘too young or too old for participation’ (Udo et al., 2019, p. 1). ‘Parentally-bereaved’ and ‘early parental death/loss’ will refer to a young person who has experienced the death of a parent (Haine et al., 2008), although we focus on those who lost a parent after the age of eight, and who are therefore likely to have some memories of that parent. The following research questions were explored:

1. What are PBYP’s experiences of grief following early parental death?
2. What aspects facilitate or impede support for PBYP following early parental death?
3. How do PBYP cope following early parental death?
4. How useful is the DPM for understanding PBYP’s experiences?

## Methods

We employed a qualitative research approach using semi-structured interviews due to the highly sensitive nature of the topic being researched (Arslan et al., 2022) and to allow subjective bereavement experiences to be explored.

### Theoretical framework

This study was informed by the Dual Process Model (Stroebe & Schut, 1999, 2010). It guided the construction of the research questions and interview guide (Supplementary material), touching upon loss- and restoration-orientation, and oscillation. Loss-oriented related-questions included emotional responses, reminiscing memories, and grief engagement. Restoration-oriented related-questions included managing life changes, and oscillation-related questions included grief avoidance. The DPM was later used as an exploratory lens through which to code and interpret the data, however, it was not deterministic of the findings as analysis took a predominately inductive approach. We also drew throughout the analysis from Arnett’s (2000) concept of ‘emerging adulthood’. This is the period from around 18–25 years where young people in many industrialised countries become more independent but have not yet taken on the responsibilities usually attributed to adults. This period can be marked by instability, particularly in relation to where young people live. It is also an opportunity for young people to explore their identities. Arnett (2000) identifies three main areas through which this is achieved – love, work and world views. It is also a time in which young people are more likely to participate in risky behaviours.

### Positionality

Positionality in research refers to the researcher’s world view and the position they adopt about a research task and its social and political context. Throughout the research process a reflexive journal was used to document decisions, rationales, and personal positions and values which were likely to influence data interpretations. The first author acknowledged that her personal experience of losing her father during adolescence was likely to influence the interview and analysis. The first author disclosed her experience of early parental death pre-interview, fostering rapport. The senior author did not have experience of being parentally bereaved as a young person, and they supervised the study design, and reviewed all study documents and analysis. This provided an outsider perspective on the study, ensuring that the research was being carried out appropriately.

### Ethical considerations

Given the nature of the research topic considerable ethical care was taken. Ethical approval was provided by the University of Glasgow School of Social and Political Sciences Ethics Forum (PGT/SPS/2022/198/SOC). The study adhered to the General Data Protection Regulation (GDPR) (Regulation (EU) 2016/679). Participants were provided with a Participant Information Sheet (PIS) and consent form electronically and given opportunities to ask questions about the research. All participants were assured of anonymity and confidentiality unless they or someone else was at risk of harm. Pseudonyms were used to protect participants’ identities. Aware of the highly sensitive nature of the topic, we anticipated that participants might express a degree of emotional distress. All participants were made aware of the topics which would be covered in the PIS. A signposting document on bereavement support services, information, and helplines was provided. Whilst conducting the interviews, the first author was sensitive to changes in body language and facial expressions. If signs of distress were expressed, participants were given the opportunity to move on to a different topic, take a break or stop the interview. A protocol was in place to support any participant who indicated they were at risk of harm during the interview.

### Recruitment

We invited young adults aged 18–25 in the UK who had experienced the death of a biological or adoptive parent more than six months ago and were aged eight or older at the time of the death. The World Health Organization defines young people as anyone between the ages of 10–24. We allowed an additional year up to 25 to account for our criterion that a bereavement needed to have been experienced more than six months previously. Six months post-bereavement has been identified as a suitable timeframe for carrying out this type of research (Maciejewski et al., 2007). Due to University ethical approval regulations for student projects only covering young people over 18 years, this was the lower limit for participants. We recruited participants using a social media advert post on Instagram and Facebook. Interested participants contacted the first author who emailed them a consent form, PIS, privacy notice and signposting document.

### Data collection

The first author conducted nine semi-structured interviews each lasting approximately one hour. The interviews addressed the following topics: experiences of grief, support and coping strategies (see Supplementary material), and were conducted remotely between May-June 2022 over Microsoft Teams. They were video- and audio-recorded. Participants received a £10 gift voucher post-interview to thank them for their participation.

### Analysis

We transcribed recordings verbatim and employed thematic analysis, following Braun and Clark (2022), supported through NVivo. We used reflective analytical memos to document decisions made, promoting credibility and dependability (Groenewald, 2008). We read and re-read anonymised transcripts to ensure accuracy and establish data familiarity. The first author identified inductive codes by systematically reading the transcripts sentence-by-sentence. The first and senior author discussed these initial themes, with the senior author reading a sample of the transcripts. This process helped identify themes encapsulated in the data, in turn, allowing the gathered data to be categorised (Williams & Moser, 2019). After agreeing on these initial categories, the first author analysed the coded data by applying the DPM as a guiding exploratory lens. Extensive broad themes and subthemes relating to the research aim and questions were compared with key concepts within the DPM and the concept of emerging adulthood. Themes were finalised to ensure they appropriately reflected the analysis of participants’ accounts.

## Results

Nine young people participated – seven women and two men. Participants’ mean age was 23 years (range 21–25) and they experienced parental loss at a mean age of 16.7 years (range 10–21). Seven experienced loss from cancer, one from a heart attack, and one from suicide. All participants were living in Scotland and educated to university level.

### Grief experiences

Participants described the difficult emotions experienced following parental loss. This was in line with the DPM’s ‘emotional reactions’ of loss-orientation (Stroebe & Schut, 1999, p. 213). For participants who had lost a parent to cancer, their grief experiences began before their parents’ death, though they did not describe their emotional reactions at that point. When their parent died, participants described feelings of shock, an inability to process what had happened, disbelief and emotional numbness.

It didn’t feel real. It felt quite like I was outwith my body…I felt like I was watching something rather than being in it. (Wendy, 19 when her dad died)

I was quite numb to it at the beginning, I kind of remember that I didn’t cry. (Laura, 20 when her dad died)

This suggests a stage prior to the DPM’s loss-oriented reactions of initial intense emotions including ‘painful longing’ (Stroebe & Schut, 1999, p. 213). The DPM does not readily account for this temporary shock and numbness described.

As participants processed the reality of the loss, anger emerged.

Pure anger…How could you leave me? (Sophie, 15 when her mum died)

One participant’s intense anger pushed others away leading to further isolation.

I lashed out at everything. I pushed away everyone around me…looking back on it now, they are such obvious signs of grief but at the time I would have never known it was that. (Esther, 14 when her mum died)

Participants associated this anger with feeling abandoned, and an inability to process their emotions and grasp the profundity of their loved one’s death. There was a sense that assistance with understanding the common connection between anger and grief would have been helpful.

As part of trying to manage the emotions experienced following a parents’ death, some participants described speaking with an external person either through counselling or therapy. Participants described mixed experiences of whether this had helped them to process their emotions. For some, they described being unable to connect with the therapist or counsellor, whilst others had maintained the relationship with that person longer-term as they continued to find it helpful.

At the time I was like this must just be what therapy is like and it wasn’t really that helpful to be honest. And since that, I actually see a proper bereavement therapist now and she is amazing. (Lucy, 21 when her dad died)

Participants generally structured their narratives around how they felt about their loss at the time of the death, and now a number of years later. Some described feeling waves of sadness around notable dates and milestones (e.g. graduation, parent’s death and birthday anniversary, Father’s and Mother’s Day).

I think probably the hardest day I’ve had was actually my graduation. (Scarlett, 19 when her dad died)

I saw all the Father’s Day cards and I just had a moment where I just broke down…I just felt it all come over me. (Wendy, 19 when her dad died)

**Figure 1.**
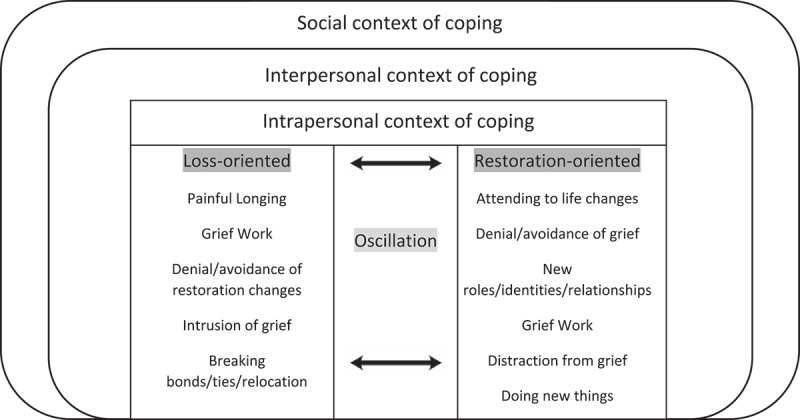
Dual Process Model framework (Stroebe & Schut, 1999) with proposed extension to account for multiple contexts of coping.

This is in line with the DPM’s loss-oriented concept of grief intrusion (Figure 1). On these unavoidable dates, a reinstated sense of loss forced grief to re-emerge. Profound emotions, including upset and yearning for the deceased, intruded (Stroebe & Schut, 2010).

Nevertheless, it was notable that four participants appeared to have drawn from their grief during this period of emerging adulthood, incorporating it into their identities as volunteers or practitioners. Despite describing avoiding the emotions that arose from their grief in the past, they had taken on roles supporting those who were bereaved through volunteer work, or employment. Grief was positioned less as an intrusion, or loss-oriented, to a means through which they could engage in restoration-oriented work for both themselves, and others. They actively shared their experiences of grief through writing for support organisations, giving presentations or participating in podcasts.

Teenage bereavement was the topic and I…ended up sharing the whole story, as much as I could at that event and it got amazing feedback based on just hearing from a young person… Another outlet for it’s like being creative, so I started making a short film about it. (Thomas, 17 when his mum died)

### Cumulative stressors

Participants described navigating their new lives as a PBYP, outlining the cumulative stressors they experienced. These surfaced in relation to the surviving parent, adult responsibilities before they were ready, bereavement practicalities and the reintegration of their new identity into their existing lives. The DPM positions the acquisition of new roles as being restoration-oriented (Figure 1), but for these participants, these new roles contributed towards their feelings of loss.

In these new roles, participants assumed tasks and made decisions previously held by the deceased, which led to further stress (Stroebe & Schut, 1999). One of these tasks was taking on responsibility for their surviving parent’s wellbeing and loneliness, a mentally and emotionally demanding endeavour which compounded their own grief. All participants with two-parent families, including one participant with divorced parents, noted this.

After he passed away, I was doing the exact same thing, but to be there for my mum…Like you know when ‘mum’s worrying’, I will do that to her…you do become really stressed. (Laura, 20 when her dad died)

One participant explicitly stated that trying to emotionally support their surviving parent to process their loss strained their relationship. Their surviving parent discarded all cherished objects relating to his deceased mother, and severely disrupted grief work (Stroebe & Schut, 1999, p. 212).

Supporting my dad ended up actually ruining our relationship…he got rid of all her stuff…all the family photos were on disks…they’re all gone. I don’t have any photos of me growing up. (Thomas, 17 when his mum died)

As described previously, emerging adulthood is conceptualised as a period during which young people have the space to experience life without being hindered by adult responsibilities. For these PBYP, this was not the reality of their lives as they emerged from adolescence. They had already assumed adult responsibilities. For some, this related to the immediate period following their parent’s death including funeral arrangements, contacting relatives and responding to friends.

There’s so much stuff to do…I was so numb…I’m sitting here picking my dad’s coffin and getting asked, do you want flowers? Like I don’t fucking know.

(Lucy, 21 when her dad died)

Me who had to phone family members and tell people at 17 because [my dad] just couldn’t face it…you’re thrown into the deep end.

(Thomas, 17 when his mum died)

More long-term challenges were practical, including household and financial management.

Learning how to do all the housework by myself and having nobody there for me. I had to cook…buy my own food…all this sort of stuff at 14 that I’d never imagined I’d have to do.

(Esther, 14 when her mum died)

I’m almost in this constant stress of do we have enough money?…My day-to-day life is a lot more grown up than I would say other people my age…I support my mum financially…I had to sell belongings.

(Wendy, 19 when her dad died)

As well as PBYP having to navigate their new status within their families, they also had to find a means through which they could reintegrate their new identity into their previous life, specifically their education and peer networks. They often immediately needed to confront the practicalities of managing coursework and exams, along with managing their grief.

I was keen to stay on top of things and not fall behind, but I just remember having several meetings with the department…It was quite cold and it wasn’t just one person that was like that…it was just very clinical and cold and these are your options.

(Esther, 14 when her mum died)

Participants described trying to navigate a course between their need for increased flexibility and understanding from others, with their frustration and resentment at being identified as different from their peers. Three participants who had lost their parent whilst in high school described seeing their names marked with a colour on the register which indicated to the teaching staff the young person’s vulnerable status. One participant described their realisation about what the colour meant.

I had a red box by [my name]…I put two and two together very fast that the red box was because this person could be ‘You need to be wary of this person. They could be like really emotional or you know, maybe need to cut them some slack’…I didn’t take to that very well.

(Daniel, 16 when his mum died)

The status of being the only PBYP in a friendship group was particularly challenging to navigate and was identified as a cause of stress. Participants described frequently that friends tended to avoid discussing or acknowledging their loss. For one participant, her feelings of alienation from her friendship group resulted in her eventually ending her connection with them.

I was with a really bitchy bunch of kids and it kind of got to the point when…they’d be like [Sophie’s] getting extra attention in school because her mum died.

(Sophie, 15 when her mum died)

Participants were unanimous in the view that shying away from the loss was an unsupportive response and obstructive in helping them cope. Peers’ avoidance disrupted friendships and isolated individuals further. This accords with the restoration-oriented component of the DPM as peers’ discomfort surrounding grief led to ‘secondary sources’ of ‘stress’ for the bereaved (Stroebe & Schut, 1999, p. 214), although the taboo around death is not specifically identified in the original model.

People get really uncomfortable when [my dad’s death is] brought up or when people don’t want to talk about it…I’ve now got to deal with your panic about the fact that my dad’s dead. That’s so unhelpful.

(Lucy, 21 when her dad died)

A few interviewees were particularly critical of peers who instead of listening, tried to resolve their grief through using clichés about loss, curtailing PBYP’s ability to heal.

When someone’s like ‘your dad’s watching over you’…I find it frustrating…people want to give you some sort of remedy or they want to give you something that’s going to fix it.

(Scarlett, 19 when her dad died)

### Valued support

Participants discussed support-systems, particularly educational facilities (schools, universities) and peers. This included emotional support, signposting, acknowledgement, and listening. Participants perceived educational facilities and peers’ actions, responses and behaviours to play a fundamental role in their coping abilities. The social context of coping is not adequately addressed in grief models, and therefore Figure 1 depicts an extension of the original DPM.

Most participants experienced particularly poor emotional support from educational institutions. Additionally, poor emotional support impeded elements of loss-orientation including grief work, grief intrusion, and breaking bonds (Figure 1). This is because it impacted their ability to receive assistance to contend with and process their loss.

The uni offer counselling, but they offer four sessions which is a bit laughable…so I guess the emotional support from the uni wasn’t there.

(Lucy, 21 when her dad died)

In contrast, a participant expressed effective emotional support from their university after their school failed to support them. However, they acknowledged that suitable emotional support provision for PBYP is not common.

I actually had a unique experience, I actually had a good experience with my uni counselling.

(Esther, 14 when her mum died)

Participants identified universities as poor at signposting PBYP to support. The lack of information provision on what bereavement services and academic support were available, and where they could find it, led participants to feel like they had wasted their time and effort. Most respondents indicated that this was a major help-seeking barrier which negatively impacted their grieving process and wellbeing outcomes.

I plucked up the courage to go into uni and ask for help…it was the wrong person…I broke down in front of this random woman…I never went to the other place because that took a lot.

(Scarlett, 19 when her dad died)

Participants placed significance on the need for peers to recognise their loss as this helped preserve memories and commemorate their parent. This falls within the interpersonal context of coping (Figure 1), as acknowledgement from peers helped with loss-oriented coping. Peers enabling the bereaved to speak about their loved one strengthened their ‘continued relationship to the deceased person’ (Stroebe & Schut, 1999, p. 213). All participants emphasised their need and appreciation for friends who acknowledged the loss by opening conversations about their deceased parent.

I really like when people ask about [my mum]…the most helpful thing is when one of my friends would say ‘oh do you remember when your mum did this?’

(Daniel, 16 when his mum died)

In acknowledging the loss, a fundamental element towards supporting PBYP was listening. Peers listening facilitated loss-orientation as it gave the PBYP an opportunity to openly share their experience, therefore assisting with grief work, grief intrusion and continuing their relationship with their loved one (Stroebe & Schut, 1999) (Figure 1). Most participants noted a need and appreciation for friends who listen without interference. This gave them a sense of release.

I just need my friends to listen and that’s it.

(Sophie, 15 when her mum died)

### Avoidance coping mechanisms and engagement management strategies

Following parental loss, all individuals employed various mechanisms and strategies to manage their loss in their day-to-day lives. These involved avoidance-oriented coping mechanisms and engagement-oriented management strategies.

#### Avoidance coping mechanisms

Coping mechanisms adopted in the initial stages of grief were avoidance-oriented and included alcohol and drug use and distraction activities. Participants had distracted themselves from grief through self-medication and keeping busy, in line with oscillation. Although oscillation examples are vaguely detailed in the original DPB model, they refer to periods when the bereaved escapes their grief and are disengaged with stressors (Stroebe & Schut, 1999). Self-medication and distraction activities served as respite.

Drink and take drugs…I definitely did self-medicate a little bit for a little while.

(Scarlett, 19 when her dad died)

For a few respondents, they remarked that initially, self-medicating helped escape rumination and from feeling painful grief-associated emotions. Alcohol and drug use are significantly harmful in the short- and long-term which Stroebe and Schut (1999, 2010) do not adequately discuss in relation to respite activities. One male participant actively stopped drinking. He wished to reduce the risk of becoming emotional, suggesting the importance he placed on controlling displays of grief. Consequently, it hindered elements of loss-orientation including grief work and intrusion of grief (Figure 1).

I actually stopped drinking…the primary motive for that was not to get emotional.

(Daniel, 16 when his mum died)

Participants also purposely kept busy to inhibit themselves from having time or space to grieve because of the fear associated with engaging with raw, painful emotions. Most participants noted that initially, when trying to cope, they distracted themselves through socialising, work, and exercise.

I just went crazy overworking. So was going to the gym at 4:00 in the morning and then going into work…obviously I just didn’t want to think about anything.

(Lucy, 21 when her dad died)

Over time, distraction activities became harmful (e.g. burnout and spending time with toxic friends). Individuals recognised that fully avoiding their loss through distraction was ineffective, only provided temporary grief-relief, and led to poor wellbeing outcomes. Stroebe and Schut (1999) give considerably little attention to health and wellbeing outcomes arising from respite activities. They argue that periods of disengagement from grief can reinstate wellbeing, however, retrospectively, almost all participants acknowledged that their initial distraction coping techniques were unhelpful and harmful. This highlights how seemingly helpful coping strategies adopted by individuals may provide short-term restoration but lead to longer-term harm.

It was kind of a vicious cycle of almost burning yourself out. (Laura, 20 when her dad died)

#### Engagement management strategies

Participants described using engagement-oriented management strategies including creative outlets and symbolic rituals. These strategies were associated with grief-related emotions and reminiscence of their deceased parent. This effectively assisted them with their management of grief work and grief intrusion (Figure 1).

Me and my dad, we used to go to the beach a lot because that was somewhere that my mum really liked. (Sarah, 10 when her mum died)

Importantly, rather than bringing up powerful, negative emotions such as sadness, these activities seemed to enhance ‘pleasurable reminiscing’, signifying that these activities restored pleasant emotions (Stroebe & Schut, 1999, p. 213). This is considered as a loss-oriented response, however, the DPM inadequately accounts for how this might assist in fostering more positive loss-related emotions.

As time passed, creative practice played an important role in remembering activities and participants framed these activities as therapeutic in helping them cope. These meaningful activities gave participants pleasure and enabled them to engage with the loss and their relationship with their parent. Most participants noted that creative outlets including writing, tattoos, producing tribute videos, and DIY projects served as effective coping mechanisms.

I write poems and stories…I’ve written a few…it’s more an expressive thing for me…I sort of engaged with that creative side and used it to help me with my grief. (Wendy, 19 when her dad died)

I had this like urge to write a lot of things down and lots of memories that I probably had forgotten, like resurfaced and things. (Scarlett, 19 when her dad died)

Moreover, creating rituals on notable dates (e.g. parent’s birthday/anniversary, Father’s and Mother’s Day) also helped with the healing process. There was a sense that these rituals assisted participants through their grief and provided them with comfort on challenging days. Over half of the participants used annual rituals involving symbolic places and objects to reconnect them with, and allow them to remember, their parent.

We climbed a Munro…[my mum] loved sunflowers…so we always take a sunflower somewhere. (Sophie, 15 when her mum died)

## Discussion

### Grief experiences

The first research question aimed to explore PBYP’s experiences of grief following early parental death. *Grief experiences* uncovered participants’ range of emotions including shock and anger, later waves of sadness on notable dates and milestones, and the creativity that grief emotions sparked. Shock and anger are consistent with two of Kubler-Ross’s (1969) five stages of grief (denial, anger). Nonetheless, it neglects sadness which re-emerges later. Grief brought on by milestones is recognised in previous research on re-grief (Brewer & Sparkes, 2011; Chater et al., 2022), however, as graduation was not mentioned by Chater et al. (2022) as an important milestone, this extends the literature on moments where PBYP are forced to revisit their grief. As such, because PBYP lose their parent young, they face far more milestones which makes their grief experiences more challenging.

Some participants reported that their grief sparked creative expressions, such as writing, video creation, tattoos, and other forms of creative expression. These outlets served as a way for PBYP to process and express their grief, transforming their painful emotions into something tangible and constructive. Creative expression allows individuals to externalise their emotions, providing a sense of control (Buckle & Corbin Dwyer, 2021) and facilitating emotional regulation (Osowiecka, 2016). Moreover, a review of evidence on creative techniques and interventions for young people and adolescents who were parentally or sibling bereaved found that methods such as writing, drawing, drama, and commemorative rituals enhance emotional expression, facilitate cognitive shifts, and improve emotional regulation. These techniques also strengthen coping mechanisms, promote meaning-making, enhance communication and relationships, and reduce social isolation, all supporting psychological healing (Edgar-Bailey & Kress, 2010).

### Cumulative stressors vs valued support

The findings demonstrate that support for these participants was influenced by their social and interpersonal contexts. Grief occurs in the social context of relational challenges, such as a lack of emotional support and understanding. This extends Stroebe and Schut’s (2001) definition of grief as being physical, behavioural and physiological to also recognise it as social. PBYP faced cumulative stressors which made grieving more challenging. Some participants were unable to enjoy the traditionally carefree experiences of emerging adulthood, and instead had to manage increased short-term and longer-lasting responsibilities. This was compounded when their support needs remained unmet due to others’ lack of knowledge and understanding. Patterson and Rangganadhan (2010) have highlighted these issues for PBYP with educational facilities and peers. Concern for a surviving parent is consistent with previous studies too (Bergman et al., 2017; Luecken & Roubinov, 2012). This study’s participants highlighted particularly their concern for their surviving parents’ wellbeing and loneliness, as well as financial issues. These relational, as well as practical challenges, appear to undermine participants’ ability to achieve growth during a period of emerging adulthood. Participants reported a sense of unpreparedness for adult duties they faced, supporting Patterson and Rangganadhan’s (2010) finding that PBYP need practical assistance with household responsibilities. Listening and acknowledgement of their loss were identified as valued support. The importance of effective support-systems to help cope with parental loss has been well-established (e.g. Bowlby, 1980; Chater et al., 2022; Lawrence et al., 2006; Smith & Langer, 2021). Without this, it is evident that PBYP are at increased risk of poor mental and physical health outcomes (Appel et al., 2018; Hollingshaus & Smith, 2015; Luecken & Roubinov, 2012; Pham et al., 2018; Raposa et al., 2014).

### Avoidance coping mechanisms and engagement management strategies

The third research question focussed on PBYP’s coping strategies following parental death. The findings suggested that participants adopted avoidance-oriented coping mechanisms including self-medication, and distraction activities (e.g. socialising, work and exercise) particularly within the early stages of bereavement. Consistent with the literature, the current study identified alcohol and substance use as a parental bereavement outcome (e.g. Brent et al., 2009; Hamdan et al., 2013; Hoeg et al., 2017). Participants also described initially not allowing themselves time or space to grieve through distraction activities. Patterson and Rangganadhan (2010) highlighted the importance of PBYP having ‘space and time to grieve’ (p. 255), but this did not happen for the study participants. Many participants noted the harmful impact distraction activities had on their health and wellbeing outcomes over time in line with previous studies which found that suppressing emotions increases the risk of future psychological distress (e.g. Glyshaw et al., 1989; Kaplow et al., 2013; Shapiro et al., 2012).

Participants employed engagement-oriented management strategies longer-term. Participants did this through creative practices, and symbolic rituals on important dates. In doing so, they restored balance between maintaining connections and learning to live in a world without their parent. Contrary to Freud (1917), participants’ experiences did not end in a final outcome of detachment, but rather a need for continuing bonds (Klass et al., 1996). Previous research has emphasised that maintaining a connection with the deceased facilitates grief management (Conant, 1996; Webb, 2002; Worden, 2009). Additionally, it further supports the idea that preserving memories and rituals helps PBYP to construct and maintain bonds (Christ, 2000; Christ et al., 2002; Silverman & Nickman, 1996). This finding also further demonstrates problem-focussed and emotion-focussed coping (Lazarus & Folkman, 1984), as the participants engaged with loss-related emotions and continued bonds activities to help them deal with their loss actively.

### The Dual Process Model and proposed adaptations

The fourth research question asked how valuable the DPM was in understanding these PBYP’s grieving and coping processes. Our analysis revealed that while the DPM effectively outlines the oscillation between loss- and restoration-oriented coping, with some additional considerations of grief work as a restoration-oriented activity, it does not fully account for the significant impact of social and interpersonal contexts on PBYP’s grief experiences. Participants frequently described challenges stemming from inadequate support from educational institutions and peers, which added layers of complexity to their grieving process not addressed by the DPM. These experiences highlight how social and interpersonal factors can create additional stressors, impeding the oscillation between loss- and restoration-oriented coping. The need to support surviving parents emotionally and financially often forced participants into adult roles prematurely, interfering with their personal grieving processes.

Existing literature supports the significance of these external factors. Wortman and Boerner (2007) argue that traditional grief models often overlook the social context of bereavement, and Humphrey and Zimpfer (2007) emphasise the critical role of social support in the grieving process, particularly for young adults. To address these limitations, we propose an extension of the DPM that incorporates social and interpersonal contexts (see Figure 1). In this model, the outer circles represent factors such as peer relationships, educational institutions, and family dynamics. These contexts interact with the loss- and restoration-oriented processes, influencing the bereaved individual’s ability to oscillate effectively.

By integrating these external factors, the extended model offers a more comprehensive framework for understanding PBYP’s grief experiences. This adaptation acknowledges that grief does not occur in a vacuum and that social environments can significantly facilitate or hinder coping mechanisms. This theoretical advancement has practical implications. Recognising the influence of social and interpersonal contexts can inform the development of targeted support services and interventions, ensuring they address not only the individual’s internal coping processes but also the external factors impacting their grief journey.

### Limitations and strengths

This was a small-scale exploratory study carried out as a student dissertation project. Findings are therefore limited in terms of the claims they can make. Participants were recruited through a social media post which increased the likelihood of participants responding with a similar background to the first author (female, over 21, living in Scotland and university educated). It is likely that men, PBYP from elsewhere, and those who did not attend university will have different experiences from the accounts presented in this study. This internet-only recruitment approach limited the opportunity to participate in the study to those who could afford or readily access internet data. Furthermore, the online-only interview restricted access to those with sufficient technological experience and the ability to use an online video platform. The sample also only included participants who were in contact with both parents at the time their parent died. A single participant, who was adopted, discussed having to live with a family member other than their parent after their other parent had died. Experiences for young people with only one parent are likely to be very different to the experiences presented here. Finally, this sample includes only those willing to disclose their experience. It could be assumed that they were more inclined to share their experience because they were coping with their loss and were more emotionally resilient.

Despite these limitations, the semi-structured, qualitative interviews produced rich and nuanced insights into a sensitive topic on an under-researched group, PBYP. Thus, the study provided an enriched understanding of their experiences, needs and how PBYP could be best supported to decrease adverse outcomes and enhance positive adjustment. The online interviews allowed for data to be collected from young people across Scotland, rather than a single geographical location, which was an advantage given that this was a student project with limited resources. This qualitative research also had a strong theoretical framework in applying the DPM. Previously, only very few had applied the model to empirical research with PBYP (Larsen et al., 2023; Lundberg et al., 2018; Patterson et al., 2021).

## Implications and recommendations

There is indicatory evidence to suggest that parental bereavement requires greater recognition as being a significant life event and unique challenge for young people. The participants in this study highlighted inadequate support structures within educational and healthcare institutions. They also struggled in making connections with peers. Developing programmes and interventions which enhance knowledge and confidence could appropriately equip peers and staff on how to effectively support bereaved young people (Hill & O’Brien, 2021; McManus & Paul, 2019). As highlighted, our small sample of PBYP were not representative, and future work is needed with those of different educational and financial backgrounds, young men and with more young people with non-nuclear family backgrounds. Within this future work, there are opportunities for researchers to explore PBYP’s experiences theoretically, particularly our proposed extensions to the DPM, to account for the varied social contexts in which PBYP are coping with their loss.

## Conclusion

This study investigates the profound impact of parental loss on young people, highlighting their journey towards healing amidst multifaceted, painful challenges. Despite the adversity, participants learned to balance grief and adapt to their new reality. Four themes were identified through a thematic analysis of nine semi-structured interviews with PBYP: grief experiences, culmulative stressors, valued support, and coping mechanisms and management strategies. These themes, aligned with the DPM, offer a holistic account of what it means to grieve as a PBYP, offering insights into their grief, coping mechanisms, and often misunderstood needs. Given the significant number of young people affected by parental loss, further research is essential, particularly focusing on young men, longer-term health impacts, and on the role of wider, immediate social and interpersonal support as factors influencing bereavement and coping outcomes.

## Data Availability

The participants of this study did not give written consent for their data to be shared publicly, so due to the sensitive nature of the research, supporting data are not available.
